# Association Between Family History of Diabetes, Irrational Beliefs, and Health Anxiety with 10-Year Risk of Type 2 Diabetes Mellitus: the ATTICA Epidemiological Study (2002–2012)

**DOI:** 10.1007/s12529-023-10189-8

**Published:** 2023-06-15

**Authors:** Christina Vassou, Thomas Tsiampalis, Ekavi N. Georgousopoulou, Christina Chrysohoou, Mary Yannakoulia, Christos Pitsavos, Mark Cropley, Demosthenes B. Panagiotakos

**Affiliations:** 1 Department of Nutrition and Dietetics, School of Health Sciences and Education, Harokopio University, 70 Eleftheriou Venizelou Ave., Kallithea, Athens, 176 76 Greece; 2 School of Medicine Sydney, University of Notre Dame, Sydney, Australia; 3 First Cardiology Clinic, School of Medicine, University of Athens, Athens, Greece; 4 School of Psychology, University of Surrey, Guildford, UK; 5 Faculty of Health, University of Canberra, Canberra, Australia

**Keywords:** Family history of diabetes, Irrational beliefs, Health anxiety, Obesity

## Abstract

**Background:**

This study aimed to examine the relationship between family history of diabetes, irrational beliefs, and health anxiety in the development of type 2 diabetes mellitus (T2DM).

**Method:**

ATTICA is a prospective, cohort study (2002–2012). The working sample included 845 participants (18–89 years), free of diabetes at baseline. Α detailed biochemical, clinical, and lifestyle evaluation was performed, while participants’ irrational beliefs and health anxiety were assessed through the Irrational Beliefs Inventory and the Whiteley index scale, respectively. We evaluated the association between the participants’ family history of diabetes mellitus with the 10-year risk of diabetes mellitus, both in the total study’s sample and separately according to their levels of health anxiety and irrational beliefs.

**Results:**

The crude 10-year risk of T2DM was 12.9% (95%CI: 10.4, 15.4), with 191 cases of T2DM. Family history of diabetes was associated with 2.5 times higher odds (2.53, 95%CI 1.71, 3.75) of T2DM compared to those without family history. Among participants with family history of diabetes, the highest likelihood of developing T2DM, regarding their tested psychological features (i.e., low/high irrational beliefs in the entire group, low/high health anxiety in the entire group, and low/high irrational beliefs, low/high healthy anxiety), had people with high irrational beliefs, low health anxiety (OR 3.70, 95%CI 1.83, 7.48).

**Conclusions:**

The findings underline the important moderating role of irrational beliefs and health anxiety in the prevention of T2DM, among participants at increased risk of T2DM.

**Supplementary Information:**

The online version contains supplementary material available at 10.1007/s12529-023-10189-8.

## Introduction

In the past decade, the risk of type 2 diabetes (T2DM) has shown an enormous increase globally, rising medical costs and worsening patients’ quality of life [1]. According to the World Health Organization (WHO), in 2014, 8.5% of adults were diagnosed with diabetes, while in 2019, diabetes was the immediate cause of 1.5 million deaths [2]. It has been suggested that T2DM will become one of the deadliest outbreaks of chronic diseases in the twenty-first century, despite the achievements in pharmaceutical management [3–5]. Heredity plays an important role in the development of chronic diseases, including T2DM. Normally, we would expect that people with a family history of diabetes would actively engage in lifestyle prevention activities to reduce their diabetes risk [6]. However, previous findings on the impact of family history of diabetes on relatives’ behavior were contradictory. For instance, in some cases, people with a family history were more likely to report a higher perceived risk of developing the disease and indeed to pursue lifestyle changes to prevent it (eating energy-dense foods, following the “5-a-day” recommendations, etc.), but there were also cases that people with a family history of diabetes not only had significant gaps in diabetes awareness but they also made no significant changes in their lifestyle behaviors [6–8].

Consequently, although the association between family history of diabetes mellitus and risk of T2DM is well documented, the links between them, beyond the genetic predisposition, are not well understood and appreciated. During the past years, some epidemiological studies as well as systematic reviews and meta-analyses underlined the crucial role of psychological disturbances, especially depression and anxiety, on T2DM onset [9, 10]. Yet, their role, along with other psychological features, such as beliefs, needs to be further explored. The American psychologist Albert Ellis (1995) in his rational emotive behavioral therapy (REBT) theory classified two types of beliefs, rational and irrational ones [11]. Irrational beliefs are rigid, non-pragmatic, unrealistic thoughts, generated by undesirable activating events and result in negative and dysfunctional emotions (including anxiety, health anxiety, and depression) and behavioral consequences (e.g., poor lifestyle habits) [12, 13]. Therefore, cognitions (i.e., beliefs), which are formed by past experiences, are crucial in the development of different types of anxieties, among which is health anxiety [14]. Health anxiety (HA) or “hypochondriasis” is an excessive emotion of health-related fear of having a serious disease that persists despite adequate medical assurance [14, 15]. Under specific criteria, health anxiety can be considered a mental disorder [16].

In a prior research study, we highlighted that individuals with high levels of irrational beliefs, who also had high levels of anxiety symptoms, had an excess risk of developing T2DM [18]. Furthermore, the cognitive theory assumes that health anxiety is triggered by external or internal health-related negative events, such as a serious illness or family medical history, which has also been a strong predisposition factor for physical conditions such as metabolic abnormalities and obesity [18–21]. Therefore, based on supporting evidence about the role of irrational beliefs and general anxiety in T2DM risk, the relationship between family medical history and health anxiety, and REBT theory, we proposed that individuals who have a family history of diabetes (i.e., experience undesirable activating event) (A), for which they may have irrational beliefs (B), that may contribute to health anxiety (i.e., emotional disturbance) and behavioral dysfunctional consequences (C), would have an increased likelihood to develop T2DM [2, 12, 13, 22]. Hence, we assumed that irrational beliefs and health anxiety if not mediate the relationship between familial history of diabetes and T2DM, they can possibly moderate (i.e., strengthen) it.

To the best of our knowledge, no previous study has assessed the synergistic role of irrational beliefs and health anxiety in the relationship between family history of diabetes and T2DM risk in the long term. Thus, the purpose of this work was to evaluate the association between irrational beliefs, health anxiety, and 10-year T2DM risk, in relation to family history of diabetes, as well as the role of various clinical factors and lifestyle behaviors, in a sample of apparently healthy adults. We hypothesized that irrational beliefs and health anxiety status would mediate or moderate the relationship between family history of diabetes and incident T2DM.

## Method

### Design

The ATTICA study is a prospective, observational cohort investigation that was initiated in 2001. The study was conducted in the province of Attica (with 78% urban and 22% rural areas), including Athens, which is a major metropolis [24]. At baseline (2001–2002), a random, multistage sampling was carried out, and 3042 apparently healthy volunteers residing in the greater metropolitan area of Athens, Greece, finally consented to participate (75% participation rate). Of the participants who signed up for the study, 1514 (49.8%) were men (mean (SD) age: 46 (13) years old) and 1528 (50.2%) were women (mean (SD) age: 45 (14) years old). Thorough clinical, lifestyle, and psychological assessments were performed by trained physicians, and per protocol, individuals with pre-existing CVD were excluded from the initial examination [24].

### Sample

Of the initially enrolled 3042 participants at baseline, a subsample of 853 participants (453 men (45 ± 13 years), and 400 women (44 ± 18 years)) agreed to participate in the psychological evaluation and form the working sample of this study. This subsample is representative of the total study’s sample as no statistically significant differences in terms of sex, age, social indicators, and T2DM risk factors profile were observed between the studied sample and the entire study’s population (all *p*-values > 0.4).

### Bioethics

The ATTICA study was approved by the Institutional Ethics (#017/1.5.2001) committee, and all participants have been informed of the aims and procedures and have agreed to participate by written informed consent.

### Baseline Measurements

#### Sociodemographic and Lifestyle Measurements

The sociodemographic and lifestyle characteristics assessed included age, sex, educational level attained, physical activity status, smoking, and dietary habits. Additional information about the methods and measurements applied has been described previously [24]. The MedDietScore, a validated and reliable tool (scale, range 0–55) designed to estimate the level of adherence to the Mediterranean diet, was administered to all participants [25, 26]*.* Higher values of this scale indicate greater adherence to the traditional Mediterranean diet, while lower values indicate greater adherence to the “Westernized” diet [27]. Current smokers were classified as individuals who smoked at least one cigarette per day during the previous year, former smokers were defined as those who had quit smoking more than a year ago, and the rest were classified as never smokers [24]. The International Physical Activity Questionnaire (IPAQ) short form was used to evaluate the level of physical activity. The IPAQ was used as an index of weekly energy expenditure based on frequency (times per week), duration (in minutes per time), and intensity of sports or other physical activity habits (in expended calories per time) [28]. Participants who did not report engaging in any physical activity were defined as sedentary, while the rest were classified as physically active [23]. The IPAQ was tested for criterion-related validity against exercise capacity with satisfactory results, as well as for reliability properties, showing good to high reliability in adults, consistent with previous studies [28].

#### Biochemical and Clinical Evaluation

Blood samples were collected from the antecubital vein between 8 and 10 am, while the subjects were seated after 12 h of fasting and alcohol abstinence. A Beckman Glucose Analyzer (Beckman Instruments, Fullerton, CA, USA) was used to measure blood glucose levels (mg/dL). Serum insulin concentrations were determined using radioimmunoassay (RIA100, Pharmacia Co., Erlangen, Germany). To comply with the American Diabetes Association definition of T2DM [29], participants with baseline blood glucose levels > 125 mg/dl or those who reported a prior diagnosis of T2DM or anti-diabetic medication use (*n* = 210) were excluded from the current analysis. People with type 1 diabetes were not included in the ATTICA study, whereas those with insulin resistance were included in the study if they were free of diabetes.

Weight, height, waist, and hip circumferences were also measured. Body mass index (BMI) was calculated as weight (in kg) divided by standing height (in square meters, m^2^), and obesity was defined as body mass index (BMI) ≥ 30.0 kg/m^2^ [30]. Waist (in cm) and hip (in cm) circumferences were also measured, and waist-to-hip (WH) and waist-to-height (WHt) ratios were calculated. An abnormal WH ratio was considered > 0.8 for women and > 1 for men, while an abnormal WHt ratio was > 0.5 for both sexes. Regarding further clinical parameters, arterial blood pressure (3 recordings) was measured after physical examination with the subject seating and resting for at least 30 min. Participants whose average blood pressure levels were ≥ 140/90 mmHg or were under antihypertensive medication were classified as hypertensive. Hypercholesterolemia was defined as total cholesterol levels > 200 mg/dL or the use of lipid-lowering agents. The intra- and inter-assay coefficients of variation of cholesterol levels did not exceed 9%.

#### Psychological Evaluation

Irrational beliefs were assessed at baseline using the Ιrrational Βeliefs Ιnventory (IBI), a brief self-report measure based on Ellis’s research [31]. The inventory contains 11 statements, each reflecting an irrational belief. These statements include topics such as worry, rigidity, need for approval, problem avoidance, and emotional irresponsibility [32]. Each item is followed by a 9-point bipolar scale ranging from “disagree” to “agree.” The scales are summed to yield a total score ranging from 0 to 88 (higher scores indicate greater severity of irrational beliefs). The IBI was designed to assess the association between the endorsement of irrational beliefs and various aspects of maladaptive emotion and behavior that have been developed within Ellis’s theoretical and applied model, which regards irrational beliefs as maladaptive [31]. Data from previous studies support the basic construct validity of this irrational beliefs scale [33]. In this study, the IBI scale had acceptable internal reliability, i.e., Cronbach’s *α* = 0.74. In the current study, participants were also classified into two categories, those with low irrational beliefs (< 52, i.e., less irrational beliefs/thoughts) and those with high irrational beliefs (≥ 52 median, i.e., frequent irrational beliefs/thoughts).

The Whiteley Index (WI) was used to assess health anxiety at baseline. The WI was developed by Pilowsky to assess health anxiety, and its items are based on clinicians’ experiences of illness characteristics of severe health anxiety [34]. The final items of the scale were selected in part due to their ability to discriminate individuals with severe health anxiety from those without [35]. The WI has been shown to have high test–retest reliability (*r* = 0.81) and convergent validity [35]. For the present study, the WI scale showed acceptable internal reliability, i.e., Cronbach’s *α* = 0.74. Moreover, for this study, participants were also classified into two categories, those having low levels of health anxiety (< 4, i.e., low health anxiety) and those having clinically meaningful symptoms of health anxiety (≥ 4 median).

### Ten-Year Follow-Up Examination

The ATTICA Study’s 10-year follow-up (mean follow-up duration: 8.41 years) took place between 2011 and 2012. Of the initially enrolled 3042 participants at baseline, 2583 participated at follow-up examination (85% participation rate; of those lost to follow-up, *n* = 224 could not be traced due to missing contact information and *n* = 235 denied participating). After also excluding participants diagnosed with T2DM at baseline (*n* = 210) and those who did not attend the psychological examination (*n* = 1528), the working sample consisted of *n* = 845 participants. Participants were initially contacted via telephone calls. In-person interviews were then conducted. Only phone calls were used to retrieve data from 35% of the participants. The researchers undertook a thorough assessment of the participants’ medical conditions using standardized procedures [24]. The re-examination included information regarding the development and management of diabetes over 10 years, among other endpoints. Diagnosis of Τ2DM was based on the American Diabetes Association (ADA) criteria, as performed at the baseline examination [29]. Participants who did not provide biological samples—those who could only be reached by telephone—were asked whether they had a medical diagnosis. There were no significant differences regarding age and sex distribution, baseline smoking habits, physical activity levels, and dietary habits between those who participated and those who lost to follow-up (all *ps* > *0.40*), which is critical for the validity of our conclusions [36].

### Statistical Methods

Categorical variables are presented as relative frequencies (%), while continuous variables are presented as mean values (standard deviation: SD). The P–P plot and the Shapiro–Wilk test were used to test for normality of the continuous characteristics’ distribution. The Pearson chi-squared test was used to investigate associations between individuals who developed diabetes and those who did not, testing their categorical characteristics, while the independent samples *t*-test was applied in case the data were continuous. Since the exact time to event (i.e., development of diabetes) was unknown, univariable and multivariable logistic regression was used to estimate odds ratios (ORs) and 95% confidence intervals (95% CI), both in the total sample and separately according to the participants’ level of health anxiety and irrational beliefs, assessing the association between the family history of diabetes mellitus and the 10-year risk of T2DM. The Hosmer–Lemeshow test was applied to assess the goodness of fit of the models. To assess the performance of the models, the –2loglikelihood ratio of the initial vs. the final model was also computed. Known confounders (i.e., age, sex, dietary habits, physical activity status, smoking, obesity, hypertension, hypercholesterolemia and psychological factors), as well as irrational beliefs and health anxiety, were all incorporated into the models, once collinearity was checked. An interaction analysis was also performed to identify the potential synergistic effect of participants’ irrational beliefs and health anxiety levels on the association between their family history of diabetes and the 10-year T2DM risk. Specifically, a highly significant interaction was observed between irrational beliefs and health anxiety scores with family history of diabetes on 10-year T2DM risk (*p* < 0.001). Results were adjusted for the participants’ demographic (age, sex), lifestyle (smoking status, physical activity, level of adherence to the Mediterranean diet), anthropometric (obesity), and clinical (personal history of hypertension and hypercholesterolemia) characteristics. Odds ratios (ORs) and 95% confidence intervals (95% CIs) from the nested multi-adjusted logistic regression model evaluated the association between the participants’ family history of diabetes mellitus with the 10-year risk of T2DM, both in the total study’s sample and separately according to their levels of health anxiety and irrational beliefs. As we were making multiple comparisons between irrational beliefs, health anxiety groups, we applied post hoc analyses using the Bonferroni rule to account for the inflation in type I error. All reported *p*-values are based on two-sided tests and compared to a significance level of 5%. STATA software (version 17.0, TStat S.r.I., Italy) was used for all statistical analyses.

Power analysis showed that the number of participants in the working dataset was sufficient to assess two-sided differences between study subgroups and the investigated parameters greater than 20%, reaching statistical power > 0.80 at < 0.05 probability level (*p*-value).

## Results

### Baseline Characteristics and Family History of Diabetes

From the entire sample, 13% (13% men, 12% women) reported family history of T2DM. Those who had family history of T2DM had also higher BMI (26 vs 24.5 kg/m^2^, *p* = 0.02), whereas no other associations were observed between family history of T2DM and age, sex distribution, and baseline fasting glucose, insulin, and triglycerides levels, arterial blood pressure or history of hypertension, hypercholesterolemia, IBI and WI scores (all *p*-values > 0.10; data not shown).

### Ten-Year Risk of T2DM

In total, *n* = 191 T2DM cases were observed during the follow-up period; of them, *n* = 97 (13.4%, 95%CI: 10.8, 16.0) were men, and *n* = 94 (12.4%, 95%CI: 10.1, 14.7) were women; the men-to-women ratio was approximately 1-to-1 in almost all age groups. Thus, the crude 10-year incidence rate of T2DM was 12.9% (95%CI: 10.4, 15.4) or 129 per 1.000 free of diabetes participants at baseline.

Participants who developed diabetes during the 10-year follow-up had 11.8% higher irrational beliefs score at baseline examination as compared to those who did not develop (i.e., 57 vs. 51/80) (*p* < *0.001*); in addition, they had higher health anxiety score based on the Whiteley index, compared to those who did not develop diabetes (i.e., 4.5 vs. 3.6/14) (*p* < 0.001) (Table 1).

**Table 1:** Baseline characteristics of the ATTICA study’s participants according to the 10-year diabetes risk (*n* = 845)

	**Status at 10-year follow-up**		
	**Without diabetes**	**Developed diabetes**	***p***
No. of participants	654	191	
Age (years)	44 ± 13	53 ± 11	< 0.001
Sex (% men)	181 (49)	14 (51)	0.570
Current smokers (yes%)	114 (54)	11 (52)	0.620
Physical activity (% active)	165 (43)	11 (38)	0.250
MedDietScore (0–55)	28 ± 8	24 ± 10	0.010
Family history of diabetes mellitus (%yes)	82 (20)	14 (36)	< 0.001
Fasting glucose, mg/dL	88 ± 12	95 ± 14	< 0.001
Triglycerides, mg/dl	91 ± 55	120 ± 32	< 0.001
Hypertension (% yes)	84 (27)	6 (46)	< 0.001
Hypercholesterolemia (% yes)	106 (37)	14 (56)	< 0.001
Body mass index, kg/m^2^	26 ± 4	29 ± 5	< 0.001
Waist circumference, cm	88 ± 14	98 ± 16	< 0.001
Irrational Beliefs Inventory (0–88)	51 ± 11	57 ± 9	0.001
Whiteley Index (0–14)	3.6 ± 2.6	4.5 ± 3.4	< 0.001

Results are presented as frequencies and relative frequencies within type 2 diabetes mellitus (T2DM) (*n* (%)), mean and SD

Further analysis revealed that people who developed diabetes were older and reported lower adherence to the Mediterranean diet at baseline examination (all* p*-values < 0.05). Regarding anthropometric characteristics, participants who developed diabetes during the 10-year follow-up had higher BMI and waist circumference values, as well as abnormal WH and WHt ratios (all *p*-values < 0.001); additionally, they were more likely to be predisposed to diabetes due to family status; they had a medical history of hypertension and hypercholesterolemia, as well as higher fasting glucose, insulin, and triglycerides levels (all* p*-values < 0.001). No association of incident T2DM with sex, baseline smoking habits, and physical activity level was observed (Table 1).

### Ten-Year T2DM Risk in Relation to Family History of Diabetes, Irrational Beliefs, and Health Anxiety

Since irrational beliefs were highly associated with health anxiety, as well as with family history of diabetes, we further explored the role of family history of diabetes on T2DM risk, taking into consideration irrational beliefs and health anxiety, apart from biomedical and behavioral indicators. Findings from multivariable models that evaluated the association between family history of diabetes, irrational beliefs, and health anxiety with the 10-year diabetes risk are presented in Supplementary Table 1. As depicted, after adjusting for several demographic, lifestyle, and clinical and psychological characteristics (model 6), participants with family history of diabetes were found to have approximately 2.5 times higher odds of developing T2DM during the 10-year follow-up. In addition, participants with higher levels of irrational beliefs, as well as those with higher levels of health anxiety, were found to have significantly higher odds of T2DM (model 6). In particular, 1-unit increment in the irrational beliefs scale was significantly associated with an increase by 10% in the odds of developing T2DM, while 1-unit increment in the health anxiety scale was significantly associated with an increase by 19% in the odds of developing T2DM. Furthermore, family history of diabetes remained a significant predictor of T2DM risk during the 10-year follow-up, independent of irrational beliefs and health anxiety effects (model 6).

### Baseline Characteristics in Relation to Irrational Beliefs and Health Anxiety

A highly significant correlation was observed between irrational beliefs and health anxiety (*r* = 0.931, *p* < 0.001). To better explore participants’ characteristics by irrational beliefs and health anxiety, they were classified into the following groups: (a) those with a “low” IBI score (i.e., < 52, median) and “low” WI score (< 4, median), (b) those with a “high” IBI score (i.e., above median value, 52) and “low” WI score (< 4), (c) those with a “low” IBI score (i.e., < 52, median) and “high” WI score (≥ 4), and (d) those with a “high” IBI score (≥ 52) and “high” WI score (≥ 4). Participants who had low levels of irrational beliefs and health anxiety represented 35.8% (*n* = 138) of our total sample. Participants with high levels of irrational beliefs and low levels of health anxiety represented 19.5% (*n* = 75) of the total sample. Participants with low levels of irrational beliefs and high levels of health anxiety represented 15.3% (*n* = 59), of the total sample, whereas those with high levels of irrational beliefs and health anxiety represented 29.4% (*n* = 113) of the sample.

Thus, to further explore the profile of participants’ characteristics in relation to irrational beliefs and health anxiety levels, additional analyses were conducted (Supplementary Table 2). It was observed that high irrational beliefs, high health anxiety group consisted slightly more of women. Also, participants were older, less educated, and less physically active as compared to the other three groups of participants (low irrational beliefs, low health anxiety; high irrational beliefs, low health anxiety; and low irrational beliefs, high health anxiety) (*all p*-values < 0.05). They also had higher % of obesity and higher hypertension levels (all* p*-values < 0.05), compared to the other three aforementioned groups (Supplementary Table 2). On the other hand, it was observed that participants with high irrational beliefs and low health anxiety were younger and men as compared to the other three groups (all* p*-values < 0.001) (Supplementary Table 2). No association of irrational beliefs and health anxiety with family history of diabetes and baseline hypercholesterolemia was revealed (all* p*-values > 0.05) (Supplementary Table 2).

However, we also conducted post hoc analyses in order to identify the significant differences within the different groups. Specifically, we found that age was different between the participants with high irrational beliefs, high health anxiety, and participants with high irrational beliefs, low health anxiety, as well as with those with low irrational beliefs, low health anxiety (all* p*-values < 0.001), while there was no difference between individuals with high irrational beliefs, high health anxiety, and those with low irrational beliefs, low health anxiety (all* p*-values > 0.05). Moreover, we observed that adherence to the Mediterranean diet was different only between the participants with high irrational beliefs, high health anxiety, and participants with low irrational beliefs, low health anxiety (*p* = 0.02).

### Multivariable Analysis of Irrational Beliefs and Health Anxiety and 10-Year Risk of T2DM

An interaction analysis was also performed to identify the potential synergistic effect of participants’ irrational beliefs and health anxiety levels on the association between their family history of diabetes and the 10-year T2DM risk, as presented in Table 2. Specifically, a highly significant interaction was observed between irrational beliefs and heath anxiety scores with family history of diabetes on 10-year T2DM risk (*p* < 0.001). Thus, when we focused on those with high levels of irrational beliefs, family history of diabetes was associated with 2.8 times higher odds of T2DM in the entire sample, 3.7 times higher odds of T2DM risk among those with low health anxiety, and 2.4 times higher odds among those with high health anxiety levels. On the other hand, when we focused on those with low levels of irrational beliefs, family history of diabetes was not associated with T2DM risk, regardless health anxiety levels. Moreover, when we focused on those with low levels of health anxiety, family history of diabetes was associated with approximately 2.7 times higher odds of developing T2DM in the total sample, mainly driven by those who also had high levels of irrational beliefs. Similarly, it was observed that among the participants with high levels of health anxiety, family history of diabetes was associated with 2 times higher odds of developing T2DM during the 10-year follow-up period only among those with high irrational beliefs, too.

**Table 2:** Odds ratios (ORs) and 95% confidence intervals (95% CIs) from the multi-adjusted logistic regression model evaluating the association between the participants’ family history of diabetes mellitus with the 10-year risk of diabetes mellitus, both in the total study’s sample and separately according to their levels of health anxiety and irrational beliefs

		**Total sample**	**Low irrational beliefs**	**High irrational beliefs**
	***N/cases***	*1485/191*	*593/55*	*892/136*
**Total sample**	*1485/191*	2.53 (1.71, 3.75)^**^	1.96 (0.93, 4.08)	2.81 (1.74, 4.54)^**^
***Low health anxiety***	*1025/110*	2.68 (1.63, 4.41)^**^	1.95 (0.93, 4.08)	3.70 (1.83, 7.48)^**^
***High health anxiety***	*460/82*	2.46 (1.40, 4.31)^*^	2.01 (0.94, 4.19)	2.38 (1.33, 4.27)^*^

Results are being adjusted for the participants’ demographic (age, sex), lifestyle (smoking status, physical activity, level of adherence to the Mediterranean diet), anthropometric (obesity), and clinical (personal history of hypertension and hypercholesterolemia) characteristics

*p < 0.05; **p < 0.001

## Discussion

In the present work, we evaluated the association between irrational beliefs, health anxiety, and 10-year T2DM risk, in relation to family history of diabetes, in a sample of apparently healthy adults. It was revealed that there are potential psychological factors that strengthen the direct path between family history of diabetes and T2DM. Particularly, we identified the moderating role of irrational beliefs and health anxiety between family history of diabetes and T2DM risk. Additionally, we observed that individuals with particular psychological characteristics, i.e., high irrational beliefs and low health anxiety, followed by individuals with high irrational beliefs, as well as individuals with high irrational beliefs and high health anxiety, had the greatest risk to develop T2DM when there was a background of family history of diabetes. Furthermore, underlying clinical and lifestyle factors, such as hypercholesterolemia, hypertension, and obesity, possibly mediated the relationship between family history of diabetes and T2DM. Despite the limitations of this work due to its observational nature, it conveys important public health messages, since it suggests, for the first time in the literature, the synergistic role of irrational beliefs and health anxiety in the relationship between family history of diabetes and the development of T2DM, as well as the potential underlying effect of particular clinical and behavioral factors.

Diabetes is a non-communicable disease that constitutes a priority for global health initiatives with a goal of halting its increase by 2025 [37–39]. Several studies have shown that certain lifestyle behaviors (e.g., regular physical activity, healthy eating habits, normal body composition) are linked to a decreased risk of diabetes [40, 41]. However, people with a family history of the disease do not necessarily perceive themselves to be at higher personal risk and do not take precautionary measures [40]. Risk and help-seeking behaviors are largely determined by individuals’ beliefs in chronic conditions like T2DM, while, in general, the contributing role of psychological aspects in the development of chronic conditions is attracting scientific interest over the last decades [20, 38–40, 42, 43]. Taking into consideration our main findings, it is quite interesting and unexpected that individuals who fell into the first two categories, i.e., high levels of irrational beliefs but low levels of health anxiety and high irrational beliefs in the entire group, when there is a family history of diabetes, are most at risk for developing diabetes, as irrational beliefs are usually accompanied by strong and negative emotions and emotional disturbances. However, the strong presence of irrational beliefs is likely to be sufficient to increase the likelihood of T2DM, when there is a strong predisposition, like family history of diabetes, probably due to irrational beliefs’ heavy cognitive burden, as well as their contribution to known T2DM risk factors, such as negative emotions, other emotional disturbances (e.g., anxiety and depression that were not tested in this study), unhealthy lifestyle behaviors, and clinical factors (obesity, hypertension, hypercholesterolemia) [17, 44–46].

Additionally, we further investigated each irrational beliefs and health anxiety group (i.e., low irrational beliefs, low health anxiety; high irrational beliefs, low health anxiety; low irrational beliefs, high health anxiety; high irrational beliefs, high health anxiety) characteristics, because they could potentially provide us more information in the interpretation of our results. However, the following information and interpretations are derived from descriptive statistics and with a relatively small number of participants. Hence, they should be viewed with caution as causation cannot be observed.

Regarding high irrational beliefs, low health anxiety category, which had the highest likelihood of developing T2DM, when there was a family history of diabetes, we noticed some differences compared to the other three groups, regarding social factors, such as age and sex. First, we found that it consisted mainly of young adults. Thus, even though irrational beliefs are generally established in childhood, individuals in their third decade may not be so much emotionally burdened to develop clinical health anxiety, as this disorder has a starting point at an older age [47]. Therefore, it was not possible to determine whether this group of people did not develop clinical health anxiety, or they developed it later in life. This information was lost since data were collected at only one point in time when the main part of the participants was still young to develop it.

Second, this group of people consisted only of men. Therefore, we assume that men either hid information about their feelings (low health anxiety) and presented only information about their beliefs (high irrational beliefs) or they suppressed their negative emotions to such a degree that they were burdened and influenced only by negative beliefs and possibly by other mental health problems. According to the biosocial model in motivational psychology, sex differences in emotions stem from an interaction between physical features and the social structure of the society [48]. A large body of literature has supported that men are less emotional expressive than women, probably because of cultural stereotypes that consider a man who expresses feelings as weak, powerless, uncontrollable, and impulsive [49]. From a developmental perspective, from childhood, men learn to conceal their feelings, while women learn to express them more freely and seek support when they experience a stressful event [50]. Thus, men demonstrate un-emotionality or the ability to hide emotions from others, simulating alternative and more acceptable emotional states [51]. This could be described as a type of bias that is called “intermediate beliefs” [52]. Intermediate beliefs are dysfunctional attitudes, rules, and assumptions that people follow in their lives and “protect” them from confronting their negative core beliefs and emotional pain, but they can be dysfunctional since they contribute to the perpetuation of negative thinking [52]. However, in modern societies, traditional gender roles have changed, and we cannot conclude with certainty that men’s emotions are opposed to their behaviors [53]. Nevertheless, our findings are well-founded, especially considering that in Southwestern and Southern European societies, like Greece, despite the shift from traditional attitudes, roles, and emotional responding, people remain more biased toward individuals with mental health problems and gender roles compared to other European countries [48, 54, 55].

Finally, regarding the third category, i.e., high irrational beliefs and health anxiety symptoms, the results were expected, and they are better explained by underlying behavioral and clinical factors. Stressful life events (SLEs) directly affect emotions and lifestyle behaviors, and they appear to be associated with cardio-metabolic complications and comorbidities [20]. Predisposing biological and genetic factors, as well as early life experiences, like a family member’s illness (e.g., a family history of diabetes), along with schemas and negative irrational beliefs (e.g., catastrophizing thinking), can contribute to the development of health anxiety [56]. Health anxiety substantially affects well-being as well as health behaviors and can contribute to medical illness, such as diabetes and an increased risk of dying from cardiovascular disease [56, 57]. It is well documented that anxiety promotes inflammation, oxidative stress, and dysfunctional activation of the autonomic nervous system and the hypothalamic–pituitary–adrenal axis [17, 20, 58]. The activation of the stress system (HPA and sympathetic-adreno-medullar (SAM) axes) and their interactions produce the acute changes caused by stress [37]. The stress system interacts with numerous systems including the central nervous system as well as the cardiorespiratory, the metabolic, and the immune system [37]. Repeated activation of the stress-related systems, such as in chronic stress, could provoke physiological dysregulation that can manifest as either hyperactivity or hypoactivity of the stress-related systems, a condition known as “allostatic load” [37]. Stress biomarkers, which enhance adaptation, can become maladaptive when physiological dysregulation occurs, operating in relatively severe physiological ranges both at rest (basal/circadian levels) and in response to stressful stimuli [37]. Physiological dysregulation might subsequently become a source of internal anxiety, and non-adaptive behaviors, like overeating and unhealthy eating patterns, can exacerbate the situation [37]. Anxiety triggers unhealthy dieting and eating behaviors, which may represent a strategy for managing health anxiety [57]. Unhealthy dietary habits are high in saturated fat and can promote both acute elevations of pro-inflammatory biomarkers, such as IL-6 and sustained low-grade systemic inflammation, as well as increased BMI, visceral fat mass, and hypercholesterolemia [57]. Accordingly, our study indicated that people with high irrational beliefs and health anxiety levels had low adherence to the Mediterranean diet, were obese, not physically active, and had high blood pressure. Also, in line with the literature, the regression analysis that we conducted showed that irrational beliefs and health anxiety probably strengthen the relationship between family history of diabetes and the development of T2DM, while hypercholesterolemia, hypertension, and obesity can relatively explain the process by which family history of diabetes and T2DM are related.

### Limitations

We are aware of the limitations of our research. Calculation of person-time and incidence rates could not be performed because the exact date of diabetes onset was not available. The date of the diabetes diagnosis was included instead. Relative risks for diabetes were calculated using odds ratios through multiple logistic regression analysis, which may have overestimated the true relative risk [57]. Nevertheless, it is recognized that the odds ratio is an accurate estimate of the relative risk for low-prevalence diseases. Another issue is that associations with disease risk were based solely on data collected at baseline, although several lifestyle factors such as eating habits might have altered throughout the 10-year follow-up period. Although no differences were detected between the participants in the psychological evaluation and the rest of the ATTICA study participants in terms of age, sex distribution, and SES level, the working sample is likely to be a limitation of the current study. The few significant, but meaningless differences in baseline characteristics between those who took part in the 10-year follow-up and those who agreed to participate in the psychological assessment might be considered a reporting bias. Also, even though the descriptive characteristics of the participants falling under each irrational beliefs, health anxiety category provided useful information that could provide an insight into why specific irrational beliefs, health anxiety groups importantly increased the likelihood of T2DM risk when there was a family history of diabetes, in comparison to others, they should be interpreted with caution, because of their observational nature and the relatively small number of participants included in each of the four categories. Lastly, aside from family history of diabetes, we did not adjust for genetic causative factors.

## Conclusion

Notwithstanding that family history of diabetes is a major predisposing factor in the development of T2DM, there was a need to identify previously unknown psychological parameters, along with biomedical implications. We theorize that irrational beliefs and health anxiety enhance the relationship between family history of diabetes and T2DM risk. It is critical to not simply embrace the strong genetic predisposition, but also to investigate the extent to which genetic predisposition can interact with cognitions and emotional state, thereby putting health status at a greater risk. Alongside, further work needs to be done to establish how challenging negative, irrational beliefs may prevent the development of diabetes through emotion and behavior regulation and to convey this message to international prevention campaigns, national health promotion programs, and primary and secondary prevention in clinical settings. Therefore, it is recommended that adults, especially the younger, who are at risk of T2DM because of genetic predisposition, undergo screening, particularly for irrational beliefs, and health anxiety symptomatology. Individuals who would exhibit high levels of these factors should be referred for REBT or other cognitive behavioral therapy (CBT) interventions. These interventions aim to dispute irrational beliefs while managing and reducing anxiety levels, such as health anxiety. By implementing these measures over time, healthcare professionals could assess whether there is prevention in T2DM directly, or indirectly, by regulating negative lifestyle behaviors and clinical complications associated with T2DM.

A part of our results has been previously presented as an e-Poster at the 90th European Atherosclerosis Society (EAS) Congress in 2022.

### Supplementary Information

Below is the link to the electronic supplementary material.

Supplementary file1 (DOCX 15 KB)

## Data Availability

The data that support the findings of this study are available from the ATTICA study but restrictions apply to the availability of these data, which were used under license for the current study, and so are not yet publicly available. Data are however available from the authors upon reasonable request and with permission of the ATTICA project.
