# Supplementary material for: Association Between Family History of Diabetes, Irrational Beliefs, and Health Anxiety with 10-Year Risk of Type 2 Diabetes Mellitus: the ATTICA Epidemiological Study (2002–2012)
Source: Int J Behav Med. 2023 Jun 15;31(4):516–26. doi: 10.1007/s12529-023-10189-8 (PMC11269384; doi:10.1007/s12529-023-10189-8)
Supplement: Supplementary file 1 — Supplementary file1 (DOCX 15 KB) [file 12529_2023_10189_MOESM1_ESM.docx]

| **Supplementary Table 1.** Odds Ratios (ORs) and 95% Confidence Intervals (95% CIs) from the nested multi- adjusted logistic regression models evaluating the association between the participants’ family history of diabetes mellitus, their health anxiety levels, and their irrational beliefs with the 10-year risk of diabetes mellitus in the total ATTICA study’s sample (n= 845). | | | | | | |
| --- | --- | --- | --- | --- | --- | --- |
|  | **Model 1** | **Model 2** | **Model 3** | **Model 4** | **Model 5** | **Model 6** |
| Family history of diabetes mellitus  (Yes Vs No) | 2.89  (2.00, 4.17)^**^ | 2.85  (1.98, 4.12)^**^ | 2.53  (1.71, 3.75) ^**^ | 2.51  (1.69, 3.74) ^**^ | 2.51  (1.69, 3.74) ^**^ | 2.53  (1.71, 3.75) ^**^ |
| Current smoker (Yes Vs No) | - | 0.94  (0.66, 1.35) | 1.06  (0.73, 1.55) | 1.04  (0.71, 1.53) | 1.04  (0.71, 1.53) | 1.04  (0.71, 1.53) |
| MedDietScore (per 1 unit) | - | 0.99  (0.97, 1.02) | 1.01  (0.97, 1.04) | 1.01  (0.97, 1.04) | 1.01  (0.97, 1.04) | 1.01  (0.97, 1.04) |
| Physical activity level (Active Vs Sedentary lifestyle) | - | 0.79  (0.56, 1.12) | 0.89  (0.61, 1.28) | 0.89  (0.61, 1.30) | 0.89  (0.61, 1.30) | 0.89  (0.61, 1.30) |
| Obesity (Yes Vs No) | - | - | 3.24  (2.14, 4.84) ^**^ | 3.06  (2.05, 4.58) ^**^ | 3.06  (2.05, 4.58) ^**^ | 3.06  (2.05, 4.58) ^**^ |
| Hypertension (Yes Vs No) | - | - | 1.45  (0.99, 2.11) ^*^ | 1.45  (0.99, 2.11) ^*^ | 1.45  (0.99, 2.11) ^*^ | 1.45  (0.99, 2.11) ^*^ |
| Hypercholesterolemia (Yes Vs No) | - | - | 1.60  (1.12, 2.30) ^*^ | 1.61  (1.12, 2.31) ^**^ | 1.61  (1.12, 2.31) ^*^ | 1.61  (1.12, 2.31) ^*^ |
| Irrational beliefs scale (per 1 unit) | - | - | - | - | 1.08  (1.00, 1.16) ^*^ | 1.10  (1.01, 1.22) ^*^ |
| Health Anxiety scale (per 1 unit) | - | - | - | 1.20  (1.00, 1.45) ^*^ | - | 1.19  (1.01, 1.47) ^*^ |
| All models are age and sex adjusted.  **p< 0.001, *p< 0.05). | | | | | | |

| **Supplementary 2**. Characteristics of participants with increased IBI score (≥52), who also had health anxiety symptoms (WI≥4) (n = 845). | | | | | |
| --- | --- | --- | --- | --- | --- |
|  | **Low irrational beliefs – Low health anxiety**  **(n=138)** | **High irrational beliefs –  Low health anxiety**  **(n=75)** | **Low irrational beliefs –  High health anxiety**  **(n=59)** | **High irrational beliefs – High health anxiety**  **(n=113)** | ***p*** |
| Family history of diabetes (%yes) | 39 (24) | 2 (20) | 1 (12.5) | 54 (21) | 0.639 |
| Age (years) | 42 ± 10 | 24 ± 4^*^ | 47 ± 20 | 48 ± 14 ^*^ | **<0.001** |
| Sex (%males) | 80 (50) | 5 (100^*^ ) | 8 (20^*^ ) | 101 (48) | **<0.001** |
| Education (years) | 16 ± 1 | 15.6 ± 0.5 | 14 ± 1 | 11 ± 2.5 | **<0.001** |
| Smoking status (%Current smoker) | 59 (34) | 1 (41) | 3 (50^*^ ) | 419 (43) | **0.014** |
| MedDietScore (0-55) | 27 ± 6 | 27 ± 2 | 26 ± 5 | 25 ± 7 ^*^ | **<0.001** |
| Physical activity level (% active) | 80 (75) | 5 (47^*^ ) | 4 (62) | 92 (40^*^ ) | **0.014** |
| Obesity (%yes) | 78 (49) | 2 (41) | 2 (37^*^ ) | 113 (60^*^ ) | **<0.001** |
| Hypertension (%yes) | 40 (24) | 2 (25) | 1 (25) | 46 (33^*^ ) | **0.009** |
| Hypercholesterolemia (%yes) | 54 (37) | 1 (28) | 2 (37) | 61 (40) | 0.188 |

^*^p-values<0.05, after corrected using the Bonferroni rule (comparisons made vs. Low irrational beliefs-Low health anxiety group as reference category).
